# Targeting extracellular Hsp90: A unique frontier against cancer

**DOI:** 10.3389/fmolb.2022.982593

**Published:** 2022-08-17

**Authors:** Rebecca A. Sager, Farzana Khan, Lorenzo Toneatto, SarahBeth D. Votra, Sarah J. Backe, Mark R. Woodford, Mehdi Mollapour, Dimitra Bourboulia

**Affiliations:** ^1^ Department of Urology, SUNY Upstate Medical University, Syracuse, NY, United States; ^2^ Upstate Cancer Center, SUNY Upstate Medical University, Syracuse, NY, United States; ^3^ Department of Biochemistry and Molecular Biology, SUNY Upstate Medical University, Syracuse, NY, United States; ^4^ Department of Medicine and Surgery, Vita-Salute San Raffaele University, Milan, Italy

**Keywords:** extracellular, Hsp90, ATP, co-chaperones, MMP2, TIMP2, cancer therapy

## Abstract

The molecular chaperone Heat Shock Protein-90 (Hsp90) is known to interact with over 300 client proteins as well as regulatory factors (eg. nucleotide and proteins) that facilitate execution of its role as a chaperone and, ultimately, client protein activation. Hsp90 associates transiently with these molecular modulators during an eventful chaperone cycle, resulting in acquisition of flexible structural conformations, perfectly customized to the needs of each one of its client proteins. Due to the plethora and diverse nature of proteins it supports, the Hsp90 chaperone machinery is critical for normal cellular function particularly in response to stress. In diseases such as cancer, the Hsp90 chaperone machinery is hijacked for processes which encompass many of the hallmarks of cancer, including cell growth, survival, immune response evasion, migration, invasion, and angiogenesis. Elevated levels of extracellular Hsp90 (eHsp90) enhance tumorigenesis and the potential for metastasis. eHsp90 has been considered one of the new targets in the development of anti-cancer drugs as there are various stages of cancer progression where eHsp90 function could be targeted. Our limited understanding of the regulation of the eHsp90 chaperone machinery is a major drawback for designing successful Hsp90-targeted therapies, and more research is still warranted.

## 1 Introduction

**FIGURE 1:**
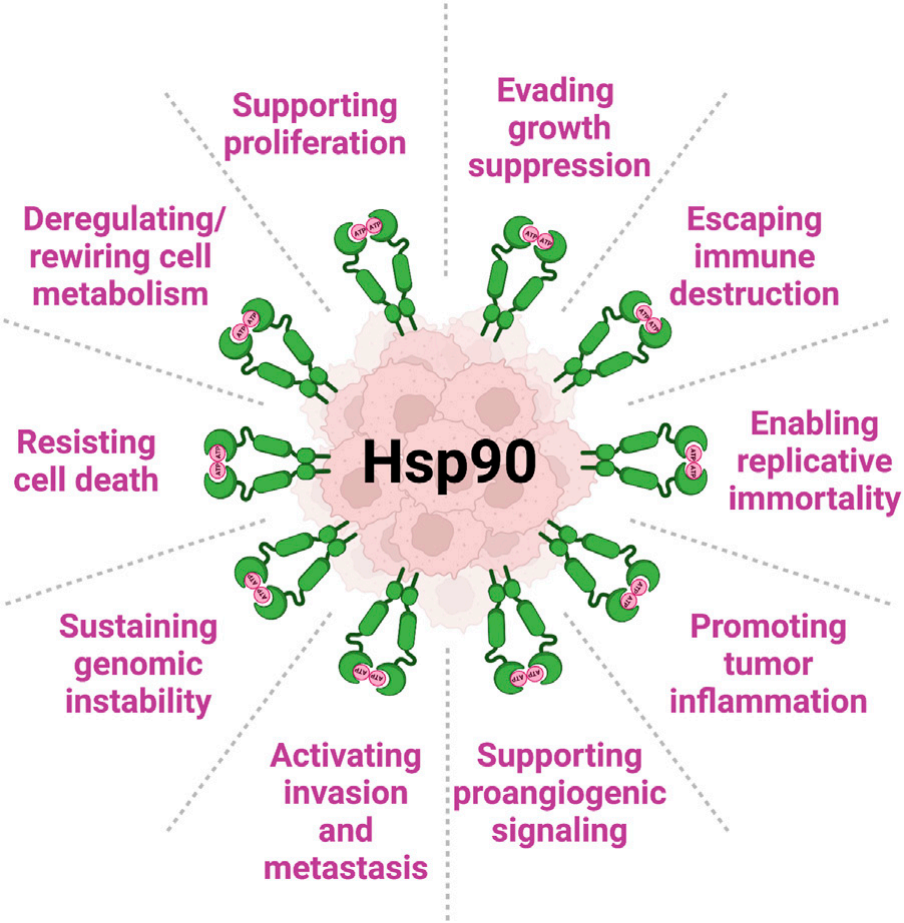
Heat shock protein 90 (Hsp90) is complicit in all Hallmarks of Cancer (per (Hanahan and Weinberg, 2011; Hanahan, 2022)). Levels of Hsp90 (both intracellular and extracellular) are significantly increased in cancer. In this review, we present several examples of Hsp90 involvement in key mechanisms of cancer initiation, development and progression.

Molecular chaperones are a large collection of proteins so named as they are known to essentially assist in maintaining protein homeostasis and, accordingly, a physiological organismal state (Hartl et al., 2011; Rebeaud et al., 2021). They have evolved to exist in all organisms, and are found both inside and outside of cells (De Maio and Vazquez, 2013; Wyatt et al., 2013; Gallotta et al., 2020). Over the lifetime of a cell or organism many types of stress pose a risk to preservation of normal cellular processes and functions to maintain homeostasis. Molecular chaperones are those proteins that help mitigate extracellular stresses and insults. They assist in the proper folding and activation of a wide array of cellular proteins and further serve as a quality control mechanism to signal irreparably damaged proteins for degradation (Schopf et al., 2017). Many of these molecular chaperones are known as heat shock proteins (HSPs), as they were initially found to be induced upon heat shock in order to assist cells in responding to thermal stress which can lead to denaturation and aggregation of proteins (Ritossa, 1962). One such family member, heat shock protein 90 (Hsp90), is an essential and ubiquitous molecular chaperone that is evolutionarily conserved (Taipale et al., 2010). There are hundreds of proteins, known as clients, that have been identified to rely on Hsp90 for their stability and function. Hsp90 is known to function largely in the final folding stage of client proteins and assists in their activation in response to various cellular signals and ligand binding (Zierer et al., 2016; Sahasrabudhe et al., 2017). Hsp90 clients span various protein classes including kinases, transcription factors, steroid hormone receptors, and many others (see https://www.picard.ch/downloads/Hsp90interactors.pdf database updated by Prof. Picard as of 02/2022) and (Echeverria et al., 2011)). In cancer, as the concentration and activity of client oncoproteins are significantly elevated, tumor cells become dependent on Hsp90 for their survival, a phenomenon known as ‘oncogene addiction’ (Workman et al., 2007). In fact, Hsp90 protein levels and chaperone function are augmented in tumors compared to the normal tissues (Figure 1).

**FIGURE 2:**
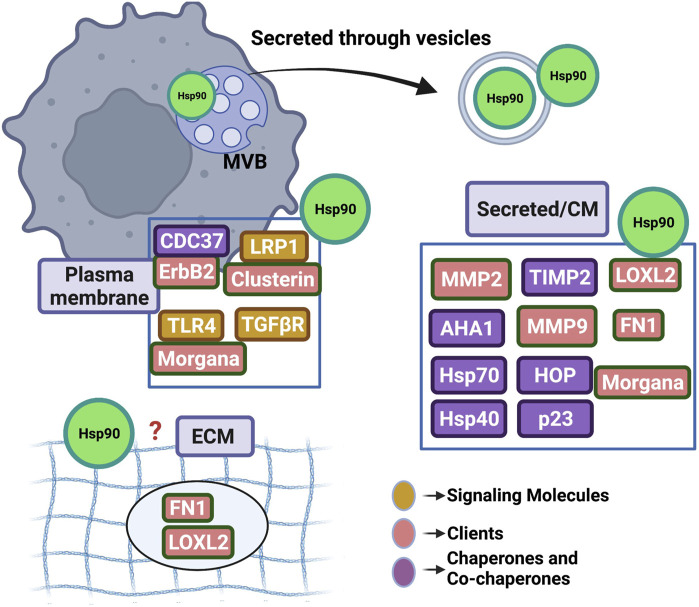
Highlighting the ubiquitous presence of Hsp90 in the extracellular space. Hsp90 (represented as green circle) has been detected at multiple sites including the outer plasma membrane, free in conditioned media (CM) and associated or enclosed into extracellular vesicles. Studies cited in this manuscript have shown eHsp90 binding directly or indirectly to several secreted protein clients, co-chaperones or signaling molecules. Although eHsp90 associates with extracellular matrix components (ECM), it remains to be found if the chaperone incorporates in the ECM structure. MVB; multivesicular body in the cytosol uptaking Hsp90 that will be released in the extracellular space following secretion.

Tumor cells actively release Hsp90 (eHsp90) into the extracellular space where it participates in the pathological multistep process of metastasis by promoting tumor cell invasion (reviewed in (Li et al., 2012; Li et al., 2013; Wong and Jay, 2016; Poggio et al., 2021)). Following over a decade-long debate on whether Hsp90 uses its ATPase activity extracellularly, researchers now point towards two possible mechanisms of action (although it is still unclear if these are mutually exclusive): eHsp90 works as a signaling molecule (ATP-independent) or as traditional chaperone (ATP-dependent) (Li et al., 2013). Furthermore, similar to the ubiquitous nature of intracellular Hsp90, eHsp90 can be found at multiple extracellular locations: anchored to the plasma membrane by binding to the ectodomains of cell surface receptors (eg. HER2, LRP1, TGFβR, TLR4) (Stellas et al., 2007; Cheng et al., 2008; Sidera et al., 2008; Stellas et al., 2010; Cheng et al., 2011; El Hamidieh et al., 2012; Garcia et al., 2016; Secli et al., 2021a; Secli et al., 2021b), in complex with components and remodelers of the extracellular matrix (eg. Fibronectin, LOXL2) (Hunter et al., 2014; Edkins, 2016; de la Mare et al., 2017; Chakraborty et al., 2020; Chakraborty and Edkins, 2021), associated with co-chaperones or client proteins following cell release (eg. TIMP2, AHA1, MMPs, Morgana) (McCready et al., 2010; Song et al., 2010; Baker-Williams et al., 2019; Secli et al., 2021a), or attached on or encapsulated in tumor released extracellular vesicles (Li et al., 2012; Tang et al., 2019; Eguchi et al., 2020) (Figure 2). One would conclude that eHsp90 role is to guard multiple sites at the tumor microenvironment in order to facilitate successful disease progression. Due to the tumor supporting function of eHsp90, it has become an attractive target for development of therapeutics. Its extracellular location lends it to non-cell permeable inhibitory mechanisms including with antibody-based treatments which have shown promise for decreasing metastasis and consequently helps avoid traditional cellular toxicities. Here, we review the role of eHsp90 in cancer and its potential as a therapeutic target.

### 2 Cytosolic Hsp90: function and regulation

In human cells there are two cytosolic isoforms of Hsp90, Hsp90α and Hsp90β, as well as organelle-specific homologs including glucose-regulated protein 94 (GRP94 or gp96) in the endoplasmic reticulum (ER) and tumor necrosis factor receptor-associated protein 1 (TRAP1) localized in the mitochondrial matrix and inter-membrane space (Csermely et al., 1998; Johnson, 2012). In healthy tissue, Hsp90β is constitutively expressed while Hsp90α expression is induced upon stress (Chang et al., 2006). Cancer cells appear to predominantly utilize the stress-inducible Hsp90α as their favored intracellular chaperone to support growth, survival, migration and invasion (Trepel et al., 2010; Zuehlke et al., 2015; Kim et al., 2019).

With regards to its structural organization, Hsp90 functions as a homodimer, and each protomer consists of a N-terminal domain where the critical ATP binding pocket is located, a middle domain which serves as the site for interaction with many clients and regulatory partners, and a C-terminal domain where the constitutive dimerization occurs (Prodromou et al., 1997a; Prodromou et al., 1997b; Meyer et al., 2003; Prodromou and Pearl, 2003; Hainzl et al., 2009). Hsp90 possesses an ATPase activity which is essential for its chaperone function, and it proceeds through a series of conformational changes known as the chaperone cycle driven by N-domain dimerization and ATP hydrolysis (Obermann et al., 1998; Panaretou et al., 1998). The chaperone function of Hsp90 and directionality of the cycle is regulated by several factors including following interaction with a group of proteins known as co-chaperones as well as by post-translational modifications (PTMs) of both Hsp90 and its co-chaperones.

Hsp90 works in concert with other molecular chaperones including those in the Hsp70 and Hsp40 family to facilitate proper folding and activation of client proteins. Traditionally, Hsp70 functions in earlier stages of client protein maturation and clients are subsequently passed to Hsp90 (Prodromou et al., 1999; Wegele et al., 2006; and reviewed in Moran Luengo et al., 2019). Co-chaperones such as the Hsp70/Hsp90-organizing protein (HOP) assist in this scaffolding process and transfer of clients between chaperones. Others, such as the activator of Hsp90 ATPase (Aha1), modulate the rate of ATP hydrolysis (Panaretou et al., 2002; Meyer et al., 2004). Co-chaperones can increase or decrease the dwell time of the chaperone in certain conformational states throughout the chaperone cycle. Co-chaperone functions can also be specific to certain client classes such as the kinase-specific co-chaperone Cdc37 (Miyata and Nishida, 2004, 2005; Vaughan et al., 2008). Chaperone function is further refined by PTMs of both Hsp90 itself as well as its co-chaperones. The recent term ‘chaperone code’ refers to the assortment of all PTMs that contribute toward unique functional profiles of the chaperone machinery at any cell state (Schopf et al., 2017; Backe et al., 2020; Truman et al., 2021; Woodford et al., 2021). The combination of co-chaperone dynamics and PTMs help refine Hsp90 chaperone function to the specific needs of individual clients at various points of time within a cell.

### 3 eHsp90 regulation: role of ATP and co-chaperones

The discovery that Hsp90 exists beyond the cellular borders was initially thought to be an artifact, secondary to release upon cell death. Hsp90 is now a well-established extracellular chaperone. Hsp90 protein sequence lacks the ‘signal peptide’ required for classical mode of protein secretion, and instead it is transported using unconventional mechanisms (Cheng et al., 2008; Santos et al., 2017). In one study, eHsp90 was shown to be truncated at its C-terminus by an unknown mechanism, removing the extreme C-terminal -EEVD docking motif that serves as an interaction site for TPR domain-containing proteins to help trap Hsp90 within the cell (Wang et al., 2009). Both Hsp90α and Hsp90β have been identified outside the cell and where specified the particular isoform has been identified throughout this review. Among others, plasma membrane translocation of Hsp90α has also been shown to depend on the PLCγ1-PKCγ pathway (Yang et al., 2014). Furthermore, while eHsp90 interacts with many surface receptors much of the eHsp90 signaling function has been found to be mediated through the cell surface receptor low density lipoprotein receptor-related protein 1 (LRP1) (Cheng et al., 2008; Gopal et al., 2011; Li et al., 2012; Tsen et al., 2013; Woodley et al., 2015).

Although the pathophysiological role of eHsp90 remains the main current focus of research, studies on the molecular mechanisms of eHsp90 regulation are limited. Does eHsp90 makes use of the extracellular nucleotide or secreted co-chaperones to function? Early studies using recombinant proteins Hsp90α and Hsp90β incubated with and without ATP showed no impact on Hsp90-mediated stabilization of client matrix metalloproteinase 2 (MMP2) *in vitro* (Song et al., 2010). Shortly after, Sims et al. showed that addition of ATP, also *in vitro*, did not enhance MMP2 processing in the presence of Hsp90 and co-chaperones (Sims et al., 2011). More recently, however, Baker-Williams et al. asked the basic question whether eHsp90 has ATPase activity (Baker-Williams et al., 2019). Hsp90 purified from the conditioned media of HEK293 cells was able to not only bind to but also to hydrolyze ATP (Baker-Williams et al., 2019). Furthermore, it has been established that extracellular ATP (eATP) levels in the tumor microenvironment (TME) exceed those in the physiological extracellular environment by 10^3^–10^4^ fold, with some recorded intratumor levels exceeding 700 µM (Pellegatti et al., 2008). Although cell death at the tumor site is significant, it does not by itself account for the increase in the levels of eATP. Indeed, it has been shown that tumors consistently upregulate several pathways for ATP efflux in the TME, such as pannexin1 (PANX-1) and P2X7 (Di Virgilio and Adinolfi, 2017). Interestingly, Hsp90 may be important for the stabilization and function of P2X7 receptor contributing to the regulation of ATP release (Migita et al., 2016).

Co-chaperones HOP (HSP70-HSP90 organizing protein), and p23 were detected in the conditioned media of breast cancer cells and formed complexes with eHsp90 (Sims et al., 2011). More recently, classical intracellular co-chaperones activator of 90 kDa heat shock protein ATPase homolog 1 (AHA1) and serine/threonine-protein phosphatase 5 (PP5) were detected in the conditioned media of HEK293 cells (Baker-Williams et al., 2019). The recently reported new co-chaperone the tissue inhibitor of metalloproteinase 2 (TIMP2) is the first identified eHsp90 co-chaperone that is secreted through the classical secretory ER/Golgi pathway. TIMP2 is a ubiquitous tumor suppressor-like protein with potent anti-angiogenic activities (Stetler-Stevenson et al., 1989; Stetler-Stevenson et al., 1990; Seo et al., 2003; Stetler-Stevenson, 2008; Bourboulia and Stetler-Stevenson, 2010; Bourboulia et al., 2011; Stetler-Stevenson et al., 2012; Bourboulia et al., 2013; Peeney et al., 2019a; Peeney et al., 2019b; Peeney et al., 2022). Originally discovered as one of the four members of the tissue inhibitors of matrix metalloproteinases (TIMPs), it is now shown to be a stress-inducible extracellular co-chaperone that increases Hsp90 binding to ATP, though inhibits its ATPase activity (Baker-Williams et al., 2019). TIMP2 is particularly important in extracellular signaling, not only it acts as an MMP inhibitor but also for regulating Hsp90-mediated chaperoning of MMP2; it helps load the client to Hsp90 and holds the protease in a transient inhibitory state while also being important for terminal inactivation of active enzyme MMP2 (Baker-Williams et al., 2019). TIMP2 can further be displaced by the co-chaperone AHA1, which allows ‘re-activation’ of MMP2 to occur and its ability to degrade ECM (Baker-Williams et al., 2019).

Post-translational regulation of intracellular Hsp90 also impacts its secretion. Secretion of Hsp90α was shown to be determined by phosphorylation status of Hsp90α-T90 where the T90A mutant could not be secreted (Wang et al., 2009). Phosphorylation of Hsp90β-S226 and -S255 in the charged linker was found to be relatively decreased in the extracellular space relative to intracellularly, and the non-phosphorylatable double alanine mutant was more efficiently secreted in K562 cells (Weidenauer and Quadroni, 2021). Furthermore, seven lysine residues were found to be acetylated and regulate the chaperoning of MMP2 in the extracellular space (Yang et al., 2008). The recent identification of secreted kinases and phosphorylation of extracellular protein substrates raises the question whether eHsp90 could also be post-translationally modified in the extracellular space (Bordoli et al., 2014; Tagliabracci et al., 2015; Sánchez-Pozo et al., 2018).

### 4 Functions of eHsp90

#### 4.1 ECM assembly

Extracellular Hsp90 helps to regulate extracellular matrix (ECM) assembly and stability, important for normal processes including embryogenesis, wound healing and cell migration. One extracellular client protein is fibronectin (FN1), a structural component of the ECM, and eHsp90 was shown to assist in incorporation of fibronectin into fibrils (Hunter et al., 2014; Chakraborty et al., 2020). Additionally, fibronectin is stress inducible; Hsp90 N-domain inhibitor treatment-induced heat shock response led to an increase in fibronectin secondary to HSF1 activity at heat shock elements in its promoter (Dhanani et al., 2017).

### 4.2 Signaling and communication

Extracellular Hsp90 present at the cell surface has many interacting partners and assists in transduction of signals between the extracellular environment and intracellular space. Membrane associated Hsp90 has also been found to play a role in the deformation of the membrane required for exosome release through an amphipathic helix in the open conformation though independent of ATPase activity (Lauwers et al., 2018). Heparin sulfate proteoglycans at the cell surface are involved in the anchoring of eHsp90 to the plasma membrane and are further involved in cancer cell migration and invasion and are required for efficient transmission of signals from eHsp90 to the intracellular space (Snigireva et al., 2019). Cell surface associated Hsp90 present on macrophages is involved in pattern recognition and cytokine response (Bzowska et al., 2017). Additionally, cell surface associated Hsp90 has been shown to be involved in intracellular signaling events associated with Kaposi’s sarcoma-associated herpesvirus infection, demonstrating how this machinery can be used in pathologic states (Qin et al., 2010).

### 4.3 Inflammation, fibrosis, and auto-immunity

While eHsp90 is important for normal ECM maintenance and wound healing there is also a careful balance which can tip towards promotion of inflammation and fibrosis that can become pathologic. Surface expression of Hsp90 on endothelial cells can also be increased by oxidative stress (Profumo et al., 2018). This has been suggested in the context of atherosclerotic plaques; eHsp90 has been seen to be the target of autoimmune responses in patients with carotid atherosclerosis, and increased eHsp90 was associated with diabetic vascular disease (Businaro et al., 2009; Profumo et al., 2018; Ding et al., 2022). Through LRP1, eHsp90 enhanced monocyte migration in the inflammatory injury associated with atherosclerosis (Ding et al., 2022). Plasma levels of Hsp90 have also been shown to be elevated in patients with systemic sclerosis and associated with increased inflammatory activity and worse lung function (Storkanova et al., 2021). eHsp90 has also been implicated in the pathogenesis of pulmonary fibrosis through activation of ER stress in fibroblasts via PI3K/Akt signaling (Zhang et al., 2021). Blockade of eHsp90 with a monoclonal antibody (mAb) was able to inhibit ER stress in both cellular and animal models of pulmonary fibrosis (Zhang et al., 2021). In the autoimmune disorder bullous pemphigoid eHsp90 was increased at the site of inflammation and autoantibodies were found to dysregulate the extracellular and intracellular distribution of Hsp90 in patients with this disease (Tukaj et al., 2013). Serum Hsp90 was also found to be increased in children with sepsis and associated with more severe features and multi-organ failure (Fitrolaki et al., 2016). Circulating Hsp90 is also increased in obesity in children and the ratio of the *a* and *ß* isoforms further associated the nonalcoholic fatty liver disease (Balanescu et al., 2019).

### 4.4 Wound healing

One of the major biological processes in which eHsp90 has been implicated is wound healing. Hsp90 was found to be secreted *via* the unconventional exosome pathway in response to TGFα stimulation and promoted epidermal and dermal cell migration through the LRP1 surface receptor (Cheng et al., 2008). Interestingly, the promotility function of eHsp90 for wound healing was independent of its ATPase activity and instead was dependent on the presence of the middle domain and charged linker (Cheng et al., 2008; Bhatia et al., 2018). Furthermore, Hsp90α secretion was found in response to HIF-1α activity as a result of hypoxia in wound healing (Li et al., 2007). Extracellular Hsp90α was found to signal through the extracellular subdomain II of LRP1 to the NPVY motif in the cytoplasmic tail of LRP1 (Tsen et al., 2013). This transduction through LRP1 mediates eHsp90α signaling to Akt-S473 phosphorylation to enhance skin cell motility and promote wound healing (Tsen et al., 2013). Normal wound healing relies on angiogenesis and secreted Hsp90 plays a role in this normal process which can also occur inappropriately in cancer. Activated endothelial cells increased secretion of Hsp90α but not Hsp90β (Song and Luo, 2010). This secreted Hsp90α localizes on the leading edge of endothelial cells to promote angiogenic activities and can also be found on blood vessels in granulation tissue of healing skin in a wound healing mouse model (Song and Luo, 2010). The functions of eHsp90 in promotion of wound healing and normal angiogenesis are then utilized by tumor cells to help promote migration and invasion.

### 5 eHsp90 and the hallmarks of cancer

In addition to the tumor progression supportive roles of intracellular Hsp90, eHsp90 also supports tumor growth and survival. eHsp90 has been implicated to play a role in many cancers and correlate with invasiveness or metastatic potential. Addition of recombinant Hsp90 to glioma and fibrosarcoma cells stimulated tumor cell invasion *in vitro* and could be blocked by anti-Hsp90 antibody, suggesting a specific extracellular effect (Snigireva et al., 2014). Elevated levels of eHsp90 have been found to correlate with metastatic potential in prostate cancer cell lines (Hance et al., 2012). Plasma Hsp90 has been developed as a biomarker in liver cancer for diagnosis as well as to evaluate efficacy of therapeutic interventions and is also elevated in advanced gastrointestinal malignancies (Fu et al., 2017; Zhang Y. et al., 2020; Wei et al., 2020). Additionally, plasma levels of Hsp90α are elevated in patients with malignant tumors compared to controls or those with benign tumors and are further elevated in those with metastatic breast cancer compared to localized disease (Wang et al., 2009). Elevated plasma Hsp90α has been employed in a comprehensive nomogram, and when combined with other markers allowed prediction of breast cancer onset and metastasis (Liu et al., 2021). Hsp90 present on the cell surface has been explored as a marker to assist in breast cancer diagnosis and margin status through fluorescent imaging, though there are technical challenges to be overcome for point-of-care translation (Crowe et al., 2017; Wang et al., 2021). There are many mechanisms by which eHsp90 supports many of the hallmarks of cancer.

### 5.1 Tumor cell migration and invasion

One study demonstrated that Hsp90α knock-out (KO) inhibited migration, invasion and metastasis without affecting growth and survival while Hsp90β KO led to tumor cell death in a breast cancer cell model (Zou et al., 2017). The tumorigenicity of eHsp90α was found to be dependent on two lysine residues, K270 and K277, which are not found in Hsp90β (Zou et al., 2017). Treatment with a monoclonal antibody, 1G6-D7, against this dual lysine region inhibited new tumor formation and expansion of already formed tumors in a mouse model (Zou et al., 2017).

**FIGURE 3:**
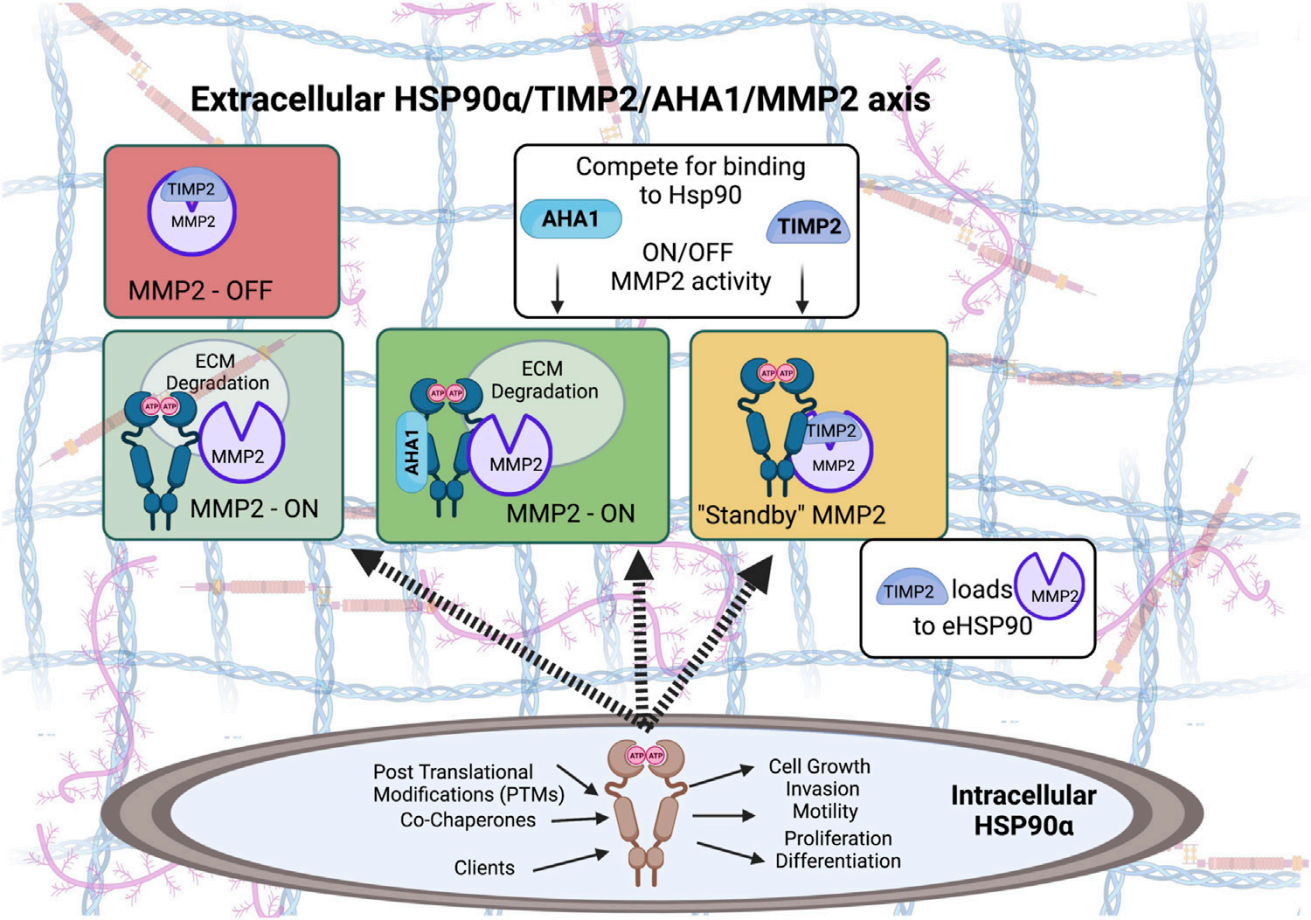
Both intracellular and extracellular Hsp90 chaperone machinery has been hijacked to support oncogenesis and tumor progression. Pro-tumorigenic, pro-invasive and pro-angiogenic secretory Matrix metalloproteinase 2 (MMP2) is a protease that degrades components of the extracellular matrix (ECM). MMP2 can function independent of eHsp90, however, MMP2 is stabilized when in complex with eHsp90α isoform. Baker-Williams et al. showed that eHsp90 chaperone function is controlled by a ‘molecular switch’ that involves two co-chaperones, TIMP2—also an endogenous MMP and angiogenesis inhibitor - and AHA1, an Hsp90 activating co-chaperone (Baker-Williams et al., 2019). TIMP2 binding to the eHsp90α/MMP2 ternary complex sets MMP2 catalytic activity in a Standby-catalytically inactive state, ready to be ‘re-activated’ when secreted AHA1 co-chaperone displaces TIMP2 from the complex.

Extracellular Hsp90-dependent invasiveness has been found to depend on several proteases important for migration and ECM remodeling including the matrix metalloproteinases (MMPs). Extracellular Hsp90α was identified in a functional proteomic screen for proteins required for invasion of human fibrosarcoma cells and found to interact with secreted MMP2 (Eustace et al., 2004). Increased MMP2 transcript levels were also found to correlate the eHsp90 surface expression in primary prostate cancer samples (Hance et al., 2012). Secreted Hsp90 promoted tumor cell invasiveness in a breast cancer model in an MMP2-dependent manner (Wang et al., 2009). Breast cancer cell migration and invasion was increased by activation of the client MMP2 following a coordinated action of eHsp90 and associated chaperones and co-chaperones including Hsp70, Hsp40, HOP, and p23 (Sims et al., 2011). Levels of catalytically active and terminally inactivated MMP2 also depends on the extracellular co-chaperone and endogenous MMP2 inhibitor TIMP2 (Figure 3) (Baker-Williams et al., 2019). Similarly, MMP3 was found to play a role in mammary cell invasion as well as normal morphogenesis through chaperoning by eHsp90β (Correia et al., 2013). Cancer cell migration has also been shown to be mediated by the extracellular client protein lysyl oxidase 2-like protein (LOXL2) (McCready et al., 2014). Another mechanism by which eHsp90α has been shown to increase cancer cell motility and invasiveness was through plasmin activation by eHsp90 in exosomes, which is another protease involved in cell migration (McCready et al., 2010).

Loss of eHsp90α present on tumor cell-secreted exosomes led to loss of tumor cell motility as well as the ability to recruit stromal cells (Tang et al., 2019). While wild-type p53 has been associated with the suppression of Hsp90α secretion, mutant p53 enhances exosome-mediated Hsp90 secretion which helps mediate survival and invasiveness of p53 mutant tumors (Zhang S. et al., 2020). In prostate cancer, extracellular vesicles (EVs) enriched with Hsp90 and other HSPs promoted EMT in normal epithelial cells, and the co-chaperone Cdc37 played a crucial role in stress-induced release of vesicles and eHsp90 to promote tumorigenesis (Eguchi et al., 2020). This was also the case in oral cancer where EVs derived from metastatic oral cancer cells promoted cell migration and invasion but triple KD of Hsp90α/Hsp90β/Cdc37 reversed these effects (Ono et al., 2020). This triple KD also reduced metastatic EV transmission into tumor associated macrophages and attenuated their cancer progression-supporting effects (Ono et al., 2020). The co-chaperone Cdc37 has also been seen associated with eHsp90 at the cell surface in complexes with oncogenic kinases including HER2 and EGFR, which also supports cancer cell invasion (Sidera et al., 2008; El Hamidieh et al., 2012).

Extracellular Hsp90 has also been shown to promote cell motility in an ERK and MMP2-dependent manner and promote a mesenchymal phenotype in prostate cancer cells (Hance et al., 2012; Wang et al., 2019). EMT in prostate cancer as a result of eHsp90 has further been shown to be the result of EZH2 expression and recruitment to the E-cadherin promoter (Nolan et al., 2015). Cancer cell migration has also been shown to be induced by the function of eHsp90 and its extracellular co-chaperone Morgana through the receptors TLR2, TLR4, and LRP1 (Secli et al., 2021a). The protein clusterin was also found to interact with and modulate eHsp90α signaling (Tian et al., 2019). Clusterin and eHsp90α functioned synergistically through LRP1 to promote EMT and migration in breast cancer cells and function through Akt, ERK, and NF-κB signaling (Tian et al., 2019). In glioblastoma, cell invasion and motility were the result of eHsp90 promoting recruitment of LRP1 to EphA2 in an Akt-dependent manner also dependent on EphA2-S897 phosphorylation (Gopal et al., 2011). Further crosstalk between eHsp90 and EphA2 was found to regulate cytoskeletal dynamics and cell morphology and attachments (Daoud et al., 2017).

### 5.2 Angiogenesis

Another hallmark of cancer in which eHsp90 plays a key role is angiogenesis. Hsp90 modulation by the co-chaperone AHA1 in endothelial cells assists in the regulation of VEGF signaling to eNOS, promoting NO production and cell migration for angiogenesis (Desjardins et al., 2012). Extracellular Hsp90α has been shown to allow breast cancer cells to survive in a hypoxic environment, which is required as tumor growth outstrips its blood supply. Secreted Hsp90α prevented hypoxia-induced cell death through an LRP1 mediated mechanism (Dong et al., 2016). Surface eHsp90 and LRP1 have been seen to be increased in response to hypoxia on tumor cells (Gopal et al., 2011). Inhibition of the eHsp90α by neutralizing antibody treatment enhanced tumor cell death under hypoxic conditions (Dong et al., 2016). Furthermore, secretion of Hsp90α was found to be dependent on HIF-1α activity in a breast cancer model (Sahu et al., 2012). HIF-1α-mediated invasion was driven by a secreted Hsp90 epitope containing the charged linker and a portion of the middle domain.

In a melanoma model, small extracellular vesicles were enriched in Hsp90 and its phosphorylated client IKKα/β in response to hypoxia (Tang et al., 2022). Delivery of this complex to cancer-associated fibroblasts activated the IKK/IκB/NF-κB/CXCL1 axis and promoted angiogenesis, highlighting a paracrine effect for eHsp90 in promoting angiogenesis (Tang et al., 2022).

Reliance of MMP2 on eHsp90 is not only important for tumor cell invasion but also for angiogenesis. MMP2 has been shown to depend on eHsp90α in an ATP-independent manner through interaction with the Hsp90 middle domain (Song et al., 2010). This was also the case in endothelial cells where eHsp90α was shown to promote angiogenesis in an MMP2-dependent manner (Song et al., 2010). The angiogenesis promoting factor macrophage migration inhibitory factor (MIF) was shown to be stabilized by Hsp90 specifically in colorectal tumor cells in a mouse model (Klemke et al., 2021). Treatment with Hsp90 inhibitor selectively interfered with the tumorigenic MIF that assisted in promoting angiogenic gene expression in tumor cells (Klemke et al., 2021). Extracellular Hsp90 also induced M2-polarized macrophages to demonstrate tumor-promoting activities including upregulation of M2 markers, phagocytosis repressors, and angiogenesis activators mediated through several signaling pathways (Fan et al., 2022).

Extracellular Hsp90 is also involved in lymphangiogenesis. In a breast cancer model increased plasma Hsp90 was associated with primary tumor lymphatic vessel density and lymph node metastasis (Hou et al., 2021). This was found to be mediated through LRP1 and downstream Akt signaling (Hou et al., 2021).

### 5.3 eHsp90 and additional cancer hallmarks

In addition to the well-studied roles in tumor cell migration, invasion, and metastasis as well as angiogenesis, eHsp90 also has additional tumor supportive roles. These include modulation of inflammatory signaling, metabolism, and cancer cell stemness. eHsp90 induced upregulation of pro-inflammatory cytokines IL-6 and IL-8 and inflammatory signaling mediator STAT3 as well as expression of fibrosis-mediator MMP3 in prostate stromal fibroblasts (Bohonowych et al., 2014). Extracellular Hsp90 and Hsp70 released from cachexia-inducing tumor cells were shown to be elevated in the plasma and induced muscle catabolism through TLR4 activation and cytokine release (Zhang et al., 2017). This led to the muscle wasting associated with cancer-related cachexia. This effect was further able to be ameliorated through inhibition of release of Hsp70 and Hsp90 via treatment with omeprazole, a proton-pump inhibitor (Liu et al., 2022). Mechanistically, this increased endolysosomal pH through vacuolar ATPase inhibition and suppressed the expression of Rab27b, which is a regulator of extracellular vesicle release (Liu et al., 2022). There are also studies linking eHsp90 to the modulation of prostate cancer stem-like cells. Extracellular Hsp90 was found to promote stemness through the upregulation of stem-like markers, plasticity, and self-renewal as well as spheroid growth (Nolan et al., 2017). Similarly, in breast cancer eHsp90 was enriched in mammosphere cultures that highly expressed markers for breast cancer stem cells, and neutralization of eHsp90 with mAb inhibited stem cell activity and ability to establish mammospheres or tumors *in vitro* and *in vivo* (Stivarou et al., 2016).

### 6 Targeting eHsp90 and its extracellular co-chaperones

When eHsp90α was identified as essential for tumor cell invasiveness, it was also demonstrated that use of cell impermeable geldanamycin beads (GA-beads) decreased MMP2 activity and invasiveness of cancer cells (Eustace et al., 2004). This indicated that eHsp90 could be directly targeted without the concerns associated with inhibiting intracellular Hsp90 (Eustace et al., 2004). Since that observation, several inhibitors have been explored and tested *in vitro* and *in vivo*, including small molecules, Hsp90 isoform specific antibodies and biologics targeting secreted chaperone-co-chaperone interactions.

Utilizing a cell-impermeable small molecule Hsp90 inhibitor (DMAG-N-oxide), eHsp90 inhibition was found to decrease tumor cell migration and ECM reorganization in cell models and decreased melanoma lung colonization in a mouse model, while not affecting intracellular Hsp90 function (Tsutsumi et al., 2008). The novel cell-impermeant inhibitor STA-12-7191 also inhibited breast cancer cell migration and was less toxic to cells than traditional intracellular Hsp90 inhibitors (McCready et al., 2014). Similarly, the pro-motility activity of tumor-secreted exosomes was blocked by anti-Hsp90 antibody (Ab) treatment and subsequently rescued by treatment with recombinant Hsp90α (Tang et al., 2019). Treatment with a monoclonal antibody targeting eHsp90α did not have a significant effect on the growth of primary tumors, however, potently prevented stromal invasion and both lymph node and distant metastasis in a breast cancer mouse model (Wang et al., 2009). More specifically, it was then also shown that treatment with the monoclonal antibody 4C5 blocked zymogens MMP2 and MMP9 processing to their active forms which concurred with disruption of their interactions with eHsp90 and inhibition of breast cancer cell metastasis to the lung (Stellas et al., 2010). Similar inhibition of lymph node and lung metastasis as well as invasiveness surrounding metastatic deposits was seen with Hsp90 mAb treatment in melanoma models (Stellas et al., 2007; Wang et al., 2009). As discussed above, eHsp90 in the plasma was associated with lymphangiogenesis and lymph node metastasis in a breast cancer model. Treatment with Hsp90α neutralizing antibody, however, was able to reduce lymphatic vessel density and sentinel lymph node metastasis (Hou et al., 2021). In fact, inhibition of Hsp90 secretion, neutralization of eHsp90, or removal the cell surface LRP1 receptor have all been shown to decrease tumor cell invasiveness and metastasis in cell and animal models (Sahu et al., 2012).

Models have also demonstrated reversal of epithelial to mesenchymal transitions (EMT) upon eHsp90 inhibition and transition back towards a less invasive epithelial phenotype. In a prostate cancer model inhibition of eHsp90 with a non-cell permeable small molecule inhibitor attenuated pro-motility signaling and consequently decreased cell migration and shifted prostate cancer cells towards a more epithelial phenotype (Hance et al., 2012).

Targeting post-translationally modified (PTM) eHsp90 has also been explored. In a breast cancer cell model pan-HDAC inhibitor treatment led to hyperacetylated eHsp90 that bound MMP2 and was associated with increased invasiveness in cell-based assays (Yang et al., 2008). Treatment with an anti-acetylated Hsp90 antibody targeting this population inhibited breast cancer cell invasion *in vitro* (Yang et al., 2008). Related, treatment of lung cancer cells with the natural compound honokiol led to hyperacetylation of Hsp90 and dissociation and degradation of MMP9 in an HDAC6-dependent manner (Pai et al., 2020).

Additionally, development of a multifunctional nanoparticle designed to target cancer stem cells was found to exert at least some of its effect through the inhibition of Hsp90 secretion, suggesting another avenue for potential therapeutics (Liu et al., 2019). Hsp90 was present on extracellular vesicles present in ascites fluid of mice harboring an aggressive T-cell lymphoma in addition to other tumor antigens in the vesicles including CD24 and Hsp70 (Menay et al., 2017). These isolated vesicles were administered to naïve mice and induced an immune response that led to rejection of tumor challenges, providing evidence for the use of exosomes to develop antitumor vaccines (Menay et al., 2017). This highlights the potential role of eHsp90 in cancer therapeutics beyond targeting Hsp90 directly.

A role for targeting eHsp90 co-chaperones and chaperone:co-chaperone complexes has also shown promise. Hsp90 has been seen to associate with co-chaperone AHA1 in secretory vesicles and at the leading edge of migrating cells, associated with migratory potential, and this complex is disrupted and the components redistributed within the cytoplasm in response to C-domain inhibitor treatment (Ghosh et al., 2015). Targeting the extracellular co-chaperones AHA1 or TIMP2 altered the ability of fibrosarcoma cells to degrade ECM (Baker-Williams et al., 2019). In fact, mechanistic and preclinical studies have established TIMP2 as a promising anti-cancer biomolecule, currently under notable preclinical development against a wide variety of human cancers (Bourboulia et al., 2012; Chowdhury et al., 2017; Peeney et al., 2022). Similarly, treatment with a mAb targeting the new extracellular co-chaperone Morgana in a breast cancer model blocked cancer cell migration and inhibited metastasis as well as reduced primary tumor growth through macrophage-dependent CD8^+^ T-cell recruitment (Secli et al., 2021a).

## 7 Conclusion and future perspectives

While traditionally thought of as an intracellular chaperone, it is now well-established that Hsp90 is ubiquitous in the extracellular space of tumor cells and supports a tumor promoting environment. This eHsp90 interacts with many surface receptors, directly and indirectly, and facilitates communication between the tumor microenvironment and the intracellular compartment. Furthermore, through chaperoning of proteins such as MMP2 and Fibronectin, eHsp90 plays a crucial role in ECM composition and remodeling. Through this interaction, eHsp90 regulates processes including wound healing and angiogenesis. An increase in levels of eHsp90 and Hsp90 within the plasma is associated with progression and metastasis in many cancers, and has been explored as a biomarker. Tumor cell migratory ability and invasiveness depends on eHsp90, and the function of this chaperone is therefore needed to support metastasis and tumor angiogenesis. Consequently, eHSP90 has become a target for development of therapeutics. In many respects, specifically targeting this population of eHSP90 is simple as cell-impermeable inhibitors or antibodies can be utilized. These have been successful in preclinical models to inhibit migration and invasion and decrease metastasis. Even more specific populations of eHsp90 have also been targeted using antibodies specific for PTMs as well as targeting co-chaperones. There is significant promise for targeting this extracellular population to inhibit the invasion and metastasis of various cancers while avoiding associated toxicities from inhibiting the intracellular population of this chaperone. Further work is needed to translate these potential therapies to clinical applications.
